# Float-Cast Microsieves with Elliptical Pores

**DOI:** 10.1021/acs.langmuir.4c01232

**Published:** 2024-10-21

**Authors:** Nadine Schwaar, Dominik Benke, Markus Retsch, Werner A. Goedel

**Affiliations:** † Chemnitz University of Technology, Physical Chemistry, Straße der Nationen 62, 09116 Chemnitz, Germany; ‡ University Bayreuth, Department of Chemistry, Chair of Physical Chemistry I, Universitätsstraße 30, 95447 Bayreuth, Germany; § Bavarian Polymer Institute, Bayreuth Center for Colloids and Interfaces, and Bavarian Center for Battery Technology (BayBatt), University of Bayreuth, Universitätsstraße 30, 95447 Bayreuth, Germany

## Abstract

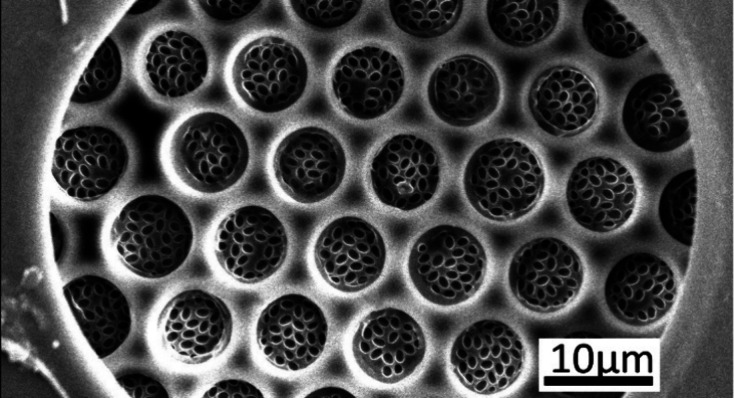

Polymeric microsieves bearing elliptical pores were successfully prepared via float-casting: a dispersion comprising nonvolatile acrylate monomers and ellipsoidal polystyrene particles was spread onto a water surface. The resulting self-organized monolayer was laterally compressed, and the monomer was photopolymerized, giving rise to a membrane comprising ellipsoidal particles laterally embedded in a 0.5 μm thin polymer membrane. The particles were dissolved, leaving behind elliptical pores. These pores had an average length of the major axis of 0.87 ± 0.1 μm and of the minor axis of 0.42 ± 0.07 μm and an aspect ratio of approximately 2. The microsieve bearing these submicrometric elliptical pores was transferred to a hierarchical structure made out of microsieves bearing circular pores of 6 μm diameter on top of a microsieve with 70 μm diameter pores. The resulting hierarchically structured microsieve had a porosity of 0.13. At a pressure difference of typically 10^3^ Pa (Reynolds number aprox. 0.002), the volumetric permeance for water was *Pe* = /*A*/Δ*p* = 0.5·10^–6^ m/s/Pa, the product viscosity·permeance is η·/*A*/Δ*p* = 0.5·10^–9^ m. This value is lower than the corresponding values of microsieves with circular pores of similar diameter produced by the same technique. The beneficial effects of higher permeance per pore caused by the elliptical shape are countered by lower porosity caused by less efficient packing of the ellipsoidal particles.

## Introduction

Microsieves, sometimes also called microfilters, microscreens, nanosieves, nanofilters, or nanoscreens, are membranes that bear a high area density of uniform through pores of micrometric or submicrometric diameters and have a thickness not significantly larger than the pore diameter.^1,2^ Materials out of which one might prepare microsieves comprise silicon nitride,^3−13^ silicon nitride coated with organic polymer,^14−17^ silicon,^17,18^ metal,^19−24^ and organic polymers.^25−43^ There are commercial suppliers for micosieves prepared via photolithography^44^ and via galvanoforming.^45^ Microsieves have been used as stencils,^12^ as support for nonporous membranes,^46−49^ as microcontactors, and as substrates for cell culture.^50^ Predominantly, they are used in filtration applications,^21,51^ like the trapping of individual particles,^52−55^ filtration with minimal filter cake build-up^56,57^ and fouling,^58,59^ for diafiltration in bioreactors,^60^ for capturing and analyzing suspended living cells^3,17,23,61^ and pathogens in drinking water,^23,51^ for membrane emulsification,^62^ filtration of emulsions,^63^ and beverage filtration.^14,8,64−68^ The uniform diameter of their pores offers a sharp size cutoff in filtration applications.^21^ The comparatively smooth surface and straight pore geometry facilitate the efficient removal of a filter cake or of fouling via cross-flow filtration,^67,68^ back-pulsing,^67−69^ and/or vibrating the sieve.^59^ The high area density of the pores of the microsieve and its small thickness give rise to a high permeance.^3,6,8,14−16,64^ The permeance and filtration properties of microsieves have been thoroughly characterized theoretically,^70,3,64,9,20,71−76,52^ via numerical simulations,^14,15,26,53−55,77−79^ and experimentally,^3,19−21,23,27,52,53,55−59,46,47,62,63,51,64−69,80^ especially with respect to their mechanical stability,^4,5,8,13−17,19,51,64,81−83^ permeance for gases,^9,14,15,26,77−79^ liquids,^3,64,16,20,75,76,80^ and dissolved polymers.^18^

Most microsieves have pores with approximately circular crossection; they may be prepared as well with hexagonal pores^8^ or with slits.^7,8,15,16,53,54,64,65,84−88^ At given sieve thickness, porosity, and pore width, microsieves with slits or elliptical pores offer a permeance higher than those with circular pores.^8,64,65,86−88^ Furthermore, if the retentate comprises spheres, they may block circular pores completely, while slit-shaped pores still allow the passage of liquid around trapped spheres though part of the slit. Thus, spheres block isolated circular pores faster and more pronounced than slits.^53,54,84−87^ Furthermore, a sphere completely blocking a circular pore is pushed toward the pore by the full transmembrane pressure difference whereas a sphere incompletely blocking a slit experiences only a hydrodynamic drag caused by the surrounding liquid flow. Thus, soft spherical objects like oil droplets or living cells larger than the pore diameter or slit width are more easily pushed through circular pores than through slits.^87^ On the other hand, removal of hard spherical particles via cross-flow or back-pulsing is easier from circular pores than from slits pores.^53,54^

Microsieves may be prepared via photolithography,^3−8,14−17,64,81^ e-beam lithography,^13^ laser interference lithography,^19,82,83^ holography,^25,26^ focused ion beam etching,^9^ ion track etching,^10^ blockcopolymer lithograpy,^11^ colloidal mask lithography,^12,70^ spontaneous formation of voids during crystallization,^18^ galvanoforming,^19−22,51^ laser ablation,^20,23,27,28^ micromolding,^29−33^ and inkjet-printing.^34^ In previous investigations, we prepared microsieves via float-casting,^89−92^ using spherical particles as spreading aids^93−97^ and porogens, and obtained microsieves with approximately circular pores.^40^ The used particles were usually silica and, in some instances, polystyrene.^43^ In the investigations here, we use ellipsoidal polystyrene particles as spreading aids and porogens in the float-casting technique to prepare microsieves bearing elliptical pores.

## Theory

**Figure 1:**
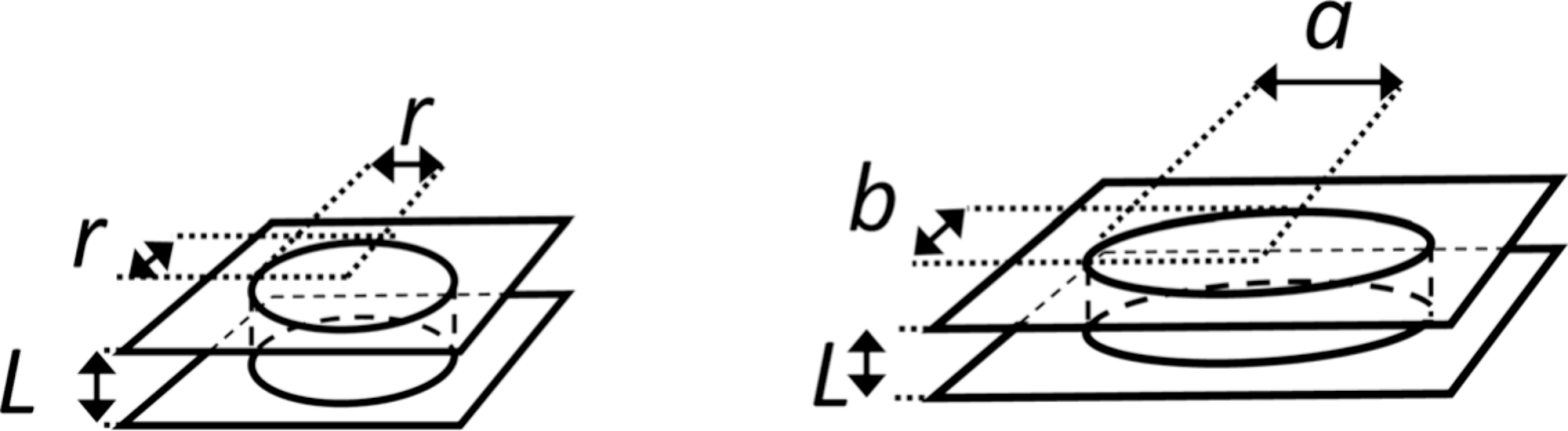
Schematic drawing of a circular pore with radius *r* and an elliptical pore with half axis *a* and *b*. *L* is the thickness of the membrane/microsieve.

**Figure 2:**
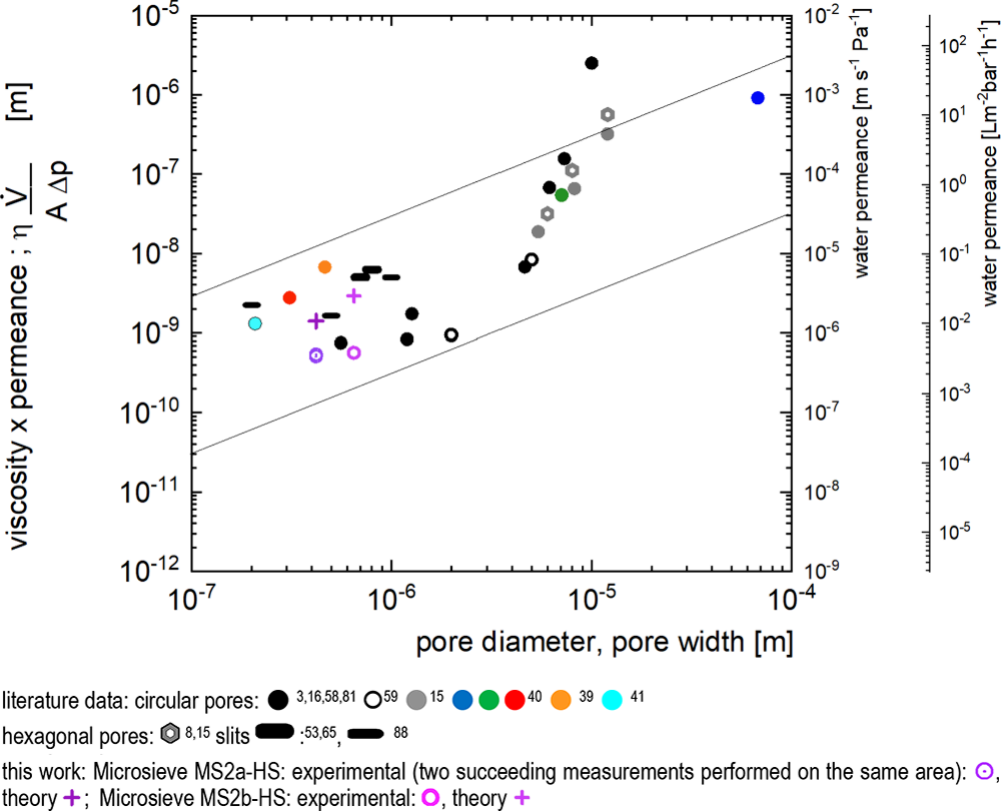
Product of viscosity·permeance of microsieves plotted versus the pore diameter or pore width, respectively; circular data points: microsieves with circular pores; hexagonal symbols: microsieves with hexagonal pores; elongated symbols: microsieves with slits or elliptical pores; literature data (indicated in the graphic).

= volumetric flow
- *A* = area of the microsieve
- Δ*p* = pressure difference
- η = dynamic viscosity
- *L* = thickness of the microsieve
- *r* = radius of a circular pore
- 2·*a* = major axis of the elliptical cross-section of the pore (see Figure 1)
- 2·*b* = minor axis of the elliptical cross-section of the pore
- *E*(ε) = complete elliptic integral of the second kind of ε
- *A*_single pore_ = π·⟨*a*·*b*⟩ = arithmetic mean area of a single pore
- *N*/*A* = areal number density of pores
- κ = *A*_single pore_·(*N*/*A*) = area of all pores/area of microsieve = porosity of the microsieve
- *Pe* = _microsieve_/*A*/Δ*p* = volumetric permeance at low Reynolds numbers

As done by the pioneers before us,^70,71,73,72,3,64,52,9,20^ we approximate the pressure drop over a single pore by summing up the contribution to the flow resistance of a tube (law of Hagen^98^ and Poiseuille^99−101^) and of the flow at the inlet and outlet (law of Sampson^102^ and Roscoe^103^ – here using the appropriate terms for a pore of elliptical cross-section).^104,103^
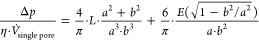
1

We follow ref (76) and take the nonuniformity of pore dimensions into account by introducing the arithmetic mean value as indicated by the angled brackets:
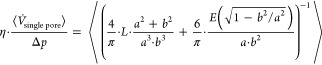
2using the relation between permeance of a microsieve and the flow through a single pore:
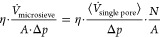
3we obtain
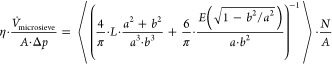
4and thus:
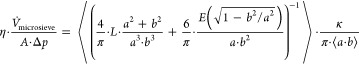
5

If all pores are uniform, we can drop the arithmetic mean and simplify eq 5 to
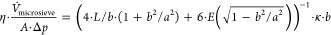
6

For circular pores *a* = *b* = *r*, (*E*(0) = π/2) eq 6 reduces to
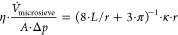
7

For very elongated elliptical pores *a* ≫ *b*, (*E*(1) = 1) eq 6 reduces to
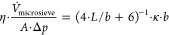
8

A comparison of eq 7 and eq 8 shows: at a given porosity, κ, and thickness, *L*, replacing circular pores of radius *r* with elongated elliptical pores of semiminor axis *b* of the same length as *r* and a semimajor axis a ≫ b in theory will roughly increase the permeance of a very thin microsieve (*L*/*b* = *L*/*r* ≈ 0) by a factor of π/2 ≈ 1.5 and the permeance of a thick microsieve (*L*/*b* ≫10) by a factor of 2. This is large enough to investigate the phenomenon from a scientific point of view. However, whether it is large enough to yield an economic or technological advantage will depend on the additional effort that may be needed to achieve the elliptical pores.

More detailed descriptions take into account the influence of the proximity of the pores;^74,75^ however, they are restricted to regular arrays of circular pores and cannot be applied here. Usually, these corrections are in the range of 10% to 20% of the permeance.

At Reynolds numbers above 4, corrections to eq 1 are needed.^3,8,9,15,77−79^ This often occurs in the case of liquids flowing through microsieves with pores of radii exceeding one micrometer and occurs even more often if, instead of a liquid, a gas is passed through the micorosieve. In the experiments carried out here, the Reynolds number is safely below this threshold.

From eq 6, eq 7, and eq 8, it becomes apparent that at a given porosity, κ, and a given ratio of *L*/*b* the theoretical permeance of a microsieve is proportional to the pore width.

Microsieves prepared and characterized by various researchers differ from each other in terms of their porosity and thickness. Thus, if we plot experimentally obtained permeances for liquids of various microsieves versus pore size, as done in Figure 2, we do not obtain a strict linear dependency. Nevertheless, the permeances can be found within a corridor with an expected slope of 1. We highlighted this corridor in Figure 2 by two straight continuous lines of slope 1, one above and one below the collection of data.

In the case of inorganic microsieves made via photolithography, the beneficial effect of slit-shaped pores in comparison to circular ones seems obvious when the corresponding data points are compared in Figure 2. In the range of pore widths a bit above and below 1 μm, reported permeances of inorganic microsieves with slit-shaped pores (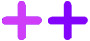) are significantly higher than the permeance of microsieves with circular pores made by the same technique by the same research group (●). However, in these examples, there is in addition a difference in porosity in favor of the microsieve with slits.

## Experimental Section

Ellipsoidal polystyrene particles were taken from the project that led to a publication of Benke, Feller, Krüsmann, Neuhöfer, Ganster, Karg, and Retsch^105^ and were prepared as described therein. In short, negatively charge-stabilized monodisperse spherical polystyrene particles (batches in the range of 0.9–1.5 μm) were embedded in a PVA matrix (Mowiol 8-88, Mw ∼ 67,000, 86.7–88.7 mol % hydrolysis, Merck, Darmstadt). The matrix was stretched uniaxially at 150 °C, a temperature above the glass transition temperature of polystyrene. The polystyrene spheres deformed to ellipsoidal particles with a defined aspect ratio that is adjustable by the degree of stretching. After recovering the ellipsoidal particles from the surrounding matrix and excessive cleanup, dispersions of ellipsoidal particles with defined aspect ratios and known mass fractions in water were obtained. The particles were subjected then to three cycles of dispersing in ethanol followed by centrifugation. Ethanol (technical grade from VWR, Dresden), isopropanol (technical grade from Brenntag, Essen), the monomer trimethylolpropane trimethacrylate (EtC(CH_2_–O–CO–CMe=CH_2_)_3_, TMPTMA, technical grade Sigma-Aldrich/Merck, Darmstadt), and the photoinitiator Omnirad TPO-L ((2,4,6-Me_3_Ph)-CO-(POOEt)-Ph, IGM-Resins B.V., Waalwijk) were added to yield a mixture comprising the following mass fractions: membrane building components: 0.016 (particles: 0.55, monomer: 0.41, initiator: 0.04) and solvent: 0.984 (ethanol: 0.503; isopropanol: 0.497). The vial containing this mixture was shaken by hand for 2 min and subjected to ultrasonication for 5 min (immersed in a water filled ultrasonic bath cleaner type USC 300T, VWR, Dresden). Part of this dispersion/solution was drawn up into a syringe bearing a cannula with a bevel tip. This tip was brought into contact with the water surface of a water-filled Langmuir trough.

The Langmuir trough (Riegler and Kirstein, Berlin) was 1 cm deep, 5 cm wide, and 32 cm long. It had two motor driven barriers with positions movable in the direction of the long axis of the trough. Both barriers were oriented perpendicular to the long axis of the trough and moved symmetrically with identical speed either inward or outward. All parts in contact with water were made from Teflon. The trough was filled to the rim with distilled water; the motor-driven barriers were moved to a distance from each other of 8 cm. Approximately 0.2 mL of the dispersion was spread. After this spreading, part of the water surface reflected light like ordinary water, and part of it was covered by patches of “gray” appearance. Five minutes after spreading, the area of the water surface between the barriers was reduced via the motor driven movable barriers with a speed of 0.4 mm per second until the patches fused into a uniform gray layer. This usually occurred at 38% of the initial area. Then, the movable barrier was stopped and kept at this position. After an additional 15 min of waiting, the surface was illuminated for 15 min with ultraviolet light emanating from a 400 W metal halide bulb (UV Curing lamp Dymax 200 flood, wavelength of emission maximum = 395 nm, illumination intensity = 0.166 W/cm^2^, Dymax, Wiesbaden). The resulting solid composite membrane that was floating on the water surface was manually scooped up from below by aluminum foil, transferred to a water filled plastic Petri dish, and stored floating. Parts of this floating membrane were scooped up manually by either copper electron microscopy grids of total diameter of 3 mm bearing 150 μm wide square openings (= 200 mesh, Plano, Wetzlar) or by polymeric hierarchical microsieves bearing 6 μm wide circular pores of approximately 1 cm total diameter and dried. The electron microscopy grids were purchased from Plano GmbH, Wetzlar. The hierarchical microsieves were prepared following the procedures of ref (40). Images of this supporting hierarchical microsieve are shown in Figure 5 top row and in Figure-S: 2 in the Supporting Information; its key properties are summarized in Table S in the Supporting Information. To dissolve the particles, the supported composite membrane was placed onto a lint-free paper wipe (Kimtech, Kimberley-Clark, Dalas, Texas). Toluene was added with a syringe dropwise onto the surface of the membrane. The position of the supported membrane on the wipe was changed by a tweezer, and the same procedure was repeated three times. This procedure yielded a membrane bearing pores not yet leading through the polymer layer but pores open only on one side while still being closed on the opposite side by a thin skin. This skin was ruptured by mounting the membrane into our setup for measuring permeance and applying a hydrostatic pressure of 1000 Pa.

**Figure 3:**
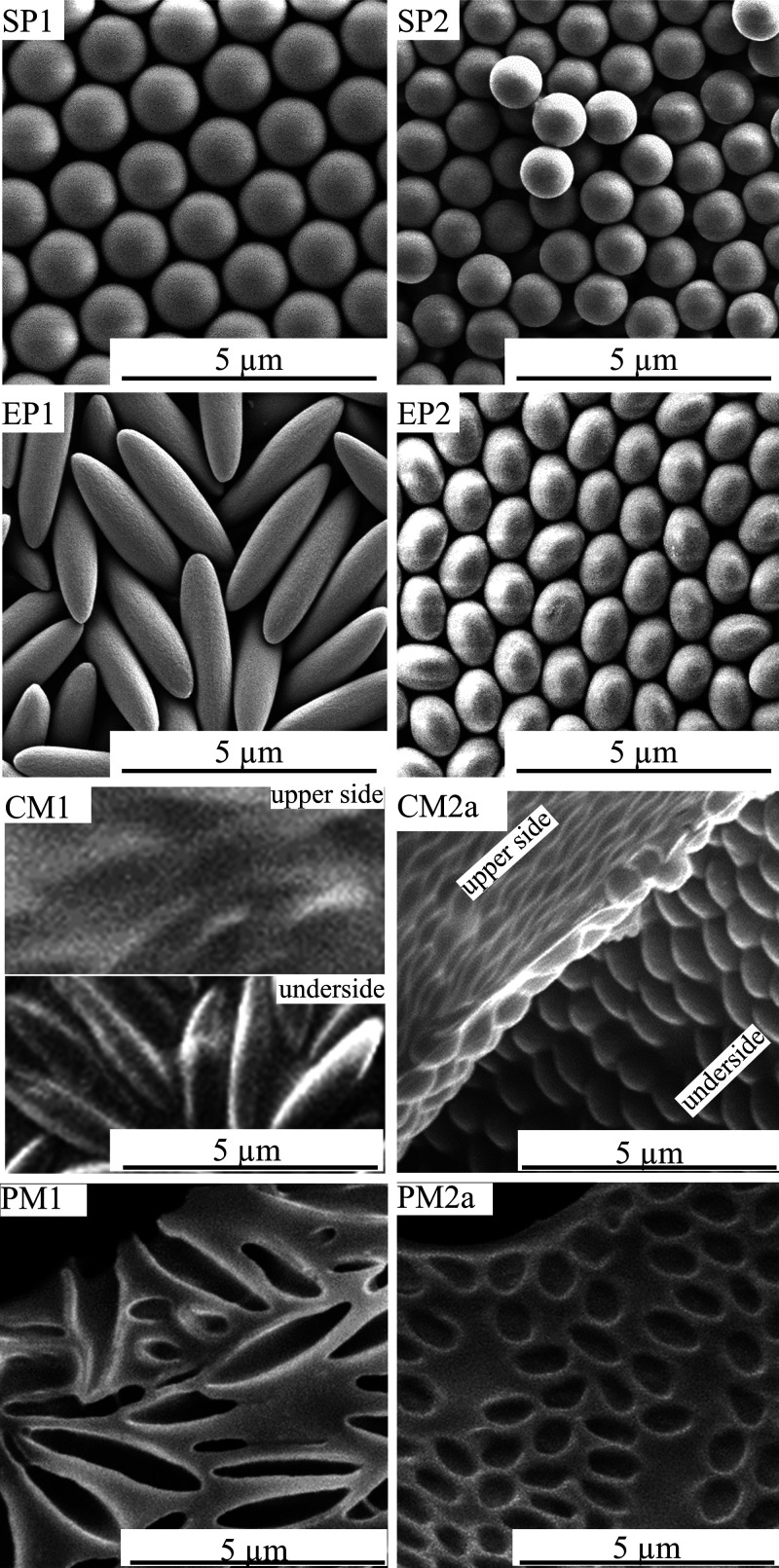
Scanning electron microscopy images of SP1, SP2: the spherical mother particles; EP1, EP2: the corresponding ellipsoidal daughter particles obtained by stretching of the mother particles; CM1, CM2: the composite membranes comprising the ellipsoidal particles generated via float-casting on a water surface; PM1, PM2a: the porous membranes obtained from the composite membranes via dissolution of the ellipsoidal particles. Zoomed-out images of the porous membranes PM1, PM2a, and PM2b can be found in Figure-S: 1 in the Supporting Information.

Dimensions of the particles and the pores were obtained via image analysis using the program ImageJ/Fiji^106,107^ using at least 50 measurements. The values reported are arithmetic means with standard deviations.

The permeance was measured using a device built from glass, similar to the one described in ref (38). The device was made from two glass plates, each bearing a central trough hole of 3 mm diameter and a glass tube connected concentrically to this hole from one side. Where connected to the glass plate, the tube was oriented vertically with respect to the plate. At a distance of 3 cm, the tube had a 90° bent. The flat side of each of the glass plates was covered with a Parafilm seal (parafilm, Bemis Inc., Nenah, Wisconsin) with a 3 mm wide circular opening in their center, placed concentrically with respect to the hole in the glass plate. The microsieve was placed between the two glass plates in such a way that both holes were aligned, and the glass plates were clamped together by steel clips. Hydrostatic pressure was applied by filling the glass tubes with water and was calculated from the height of the water column above the outlet. Permeance was calculated from permeated volume, Δ*V*, divided by time, Δ*t*, by the membrane area, *A*, and by the hydrostatic pressure Δ*p*, the latter calculated from density  = 1000 kg/m^3^, standard acceleration of gravity, *g* = 9.81 ms^–2^, and the height of the liquid above the microsieve,·*h*, according to
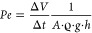
9

The experiment was run for a short enough time that *h* did not change significantly. Thus, this nonintegrated form of the equation was sufficient.

Reynolds number, *Re*, was calculated according to^108^
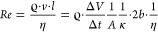
10using

the velocity of the liquid in the pore (8,15,16)

characteristic length, *l* = 2*b*

volumetric density of water ϱ_water_ = 998 kg m^–3^

dynamic viscosity of water, η_water_ = 1.00·10^–3^ Pa·s^109^

porosity, κ = κ_HS_·κ_PM_ = 0.13 [Table 4]

## Results and Discussion

**Figure 4:**
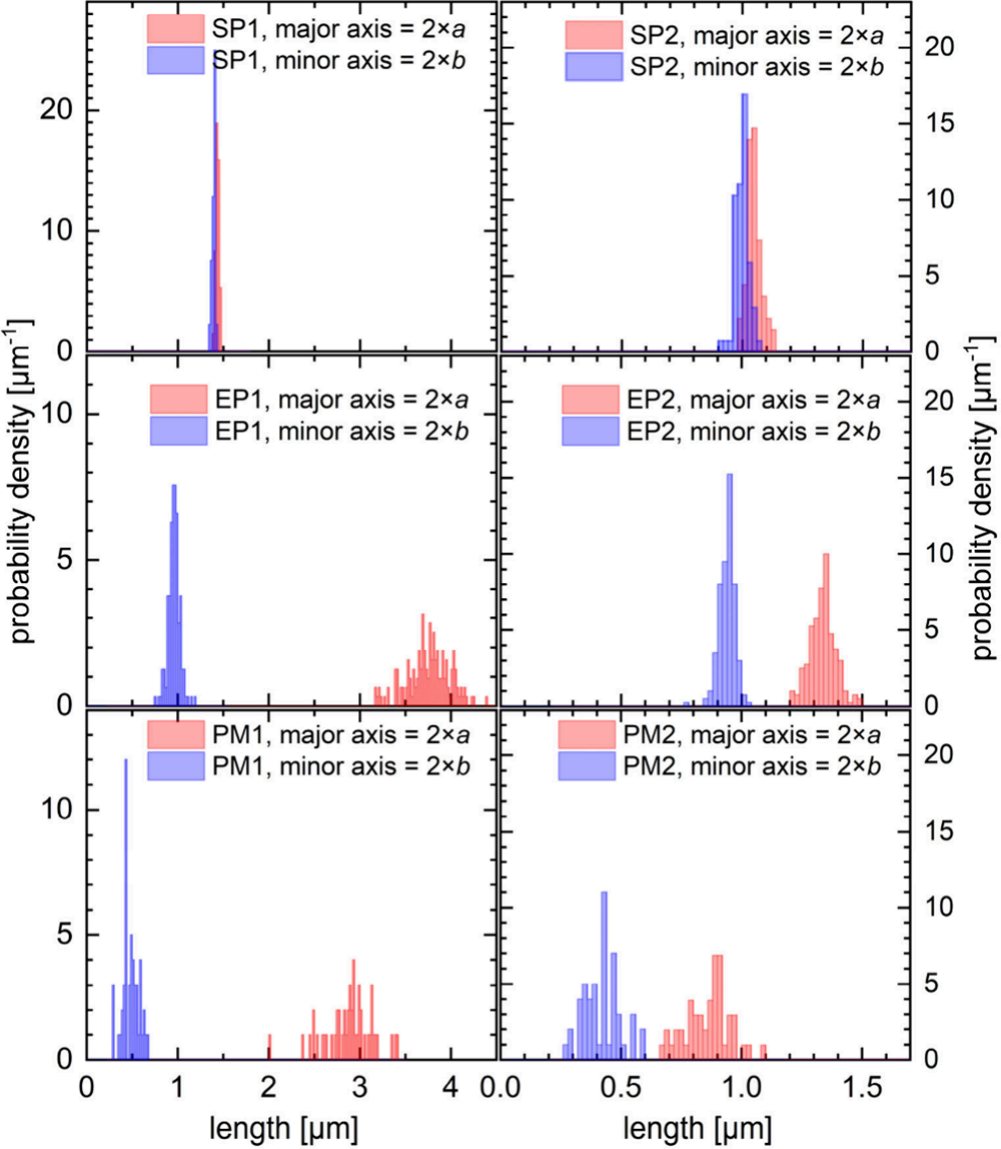
Histograms of the lengths of the major and minor axes, respectively; SP1, SP2: the spherical mother particles; EP1, EP2: the ellipsoidal daughter particles; PM1, PM2a: porous membranes made via float-casting using the ellipsoidal daughter particles.

Prolate ellipsoidal polystyrene particles (short name EP1 and EP2) were prepared via uniaxial stretching of spherical particles as described by Benke, Feller, Krüsmann, Neuhöfer, Ganster, Karg, and Retsch.^105^ Images of the spherical mother particles and ellipsoidal daughter particles are shown in the first and second rows of Figure 3. Their dimensions, as obtained from analysis of scanning electron microscopy, are depicted in the form of histograms in Figure 4 and are listed in Table 1 and Table 2.

**Table 1:** Properties of the Spherical Mother Particles

	Short Name	
	SP1	SP2
length of major axis = 2·*a* [μm]^a^	1.435 ± 0.018	1.07 ± 0.03
length of minor axis = 2·*b* [μm]^a^	1.395 ± 0.019	1.03 ± 0.03
aspect ratio = *a*/*b*^a^	1.030 ± 0.010	1.04 ± 0.02
particle volume = (4/3)·π·*a*·*b*^2^ [μm^3^]^a^	1.464 ± 0.052	0.591 ± 0.050
cross-sectional area = π·*a*·*b* [μm^2^]^a^	1.573 ± 0.037	0.861 ± 0.048
zeta-potential, [mV]	–82	–64

a Arithmetic mean value and corresponding standard deviation.

**Table 2:** Properties of the Ellipsoidal Daughter Particles

	Short Name	
	EP1	EP2
length of major axis = 2·*a* [μm]^a^	3.77 ± 0.26	1.33 ± 0.06
length of minor axis = 2·*b* [μm]^a^	0.96 ± 0.06	0.94 ± 0.03
aspect ratio = *a*/*b*^a^	3.96 ± 0.47	1.43 ± 0.07
particle volume = (4/3)·π·*a*·*b*^2^ [μm^3^]^a^	1.81 ± 0.21	0.611 ± 0.051
cross-sectional area = π·*a*·*b* [μm^2^]^a^	2.83 ± 0.22	0.978 ± 0.058
zeta-potential, [mV]	–63	–55

a Arithmetic mean value and corresponding standard deviation.

During the deformation of the particles, the volume is conserved; thus, the major half axis of the ellipsoidal daughter particles is longer than the radius of the mother particle, while the minor half axis is shorter. For the work here, we chose ellipsoidal particles with a minor axis, 2·*b*, of approximately 1 μm and aspect ratios of approximately 4 (EP1) and approximately 1.5 (EP2), respectively. As done previously with spherical particles,^35−42^ we dispersed these ellipsoidal particles in a mixture of a suitable nonwater-soluble nonvolatile multifunctional methacrylate monomer, a nonvolatile photoinitiator, and volatile organic solvents. We applied this mixture to a water surface, gave the volatile solvents time to evaporate, and solidified the monomer via photo polymerization. Thus, we generated a composite membrane composed of polystyrene particles embedded in a highly cross-linked methacrylate-based polymer. We transferred this composite membrane to 200 mesh copper grids with square shaped openings of 150 μm width. When choosing the volume ratio of monomer to particles, we aim for a monolayer of particles that are laterally embedded in a thin layer of monomer, each particle protruding out of the resulting composite membrane at the upper side into the air and at the underside into the water. If too much monomer is used, the particles usually are completely covered on the upper side of the membrane by polymer. In consequence, dissolving the particles does not yield trough pores. If not enough monomer is used, the resulting membrane is too frail. The membranes obtained after optimization of the ratio monomer to particles are shown in Figure 3, CM1 and CM2a. Afterward, we removed the ellipsoidal particles via exposure to a good solvent for polystyrene (and nonsolvent for the highly cross-linked methacrylate). leaving behind a membrane composed of cross-linked methacrylate polymer bearing pores that reflect the size and shape of the former particles (see Figure-S: 3, PM1, PM2a, and PM2b in the Supporting Information). The dimensions of the resulting pores are depicted in the form of histograms in Figure 4, and mean values are listed in Table 3.

**Table 3:** Properties of the Porous Membranes with Elliptical Pores after Dissolution of the Ellipsoidal Particles

	Short Name		
	PM1	PM2a	PM2b
thickness, *L* [μm]	0.3 ± 0.09	0.5 ± 0.05	0.33 ± 0.05
length of major axis of a pore = 2·*a* [μm]^a^	2.89 ± 0.28	0.87 ± 0.1	0.93 ± 0,08
length of minor axis of a pore = 2·*b* [μm]^a^	0.48 ± 0.09	0.42 ± 0.07	0.65 ± 0.07
aspect ratio of a pore = *a*/*b*^a^	6.22 ± 1.33	2.12 ± 0.47	1.45 ± 0.18
area of a pore = π·*a*·*b* [μm^2^]^a^	1.10 ± 0.25	0.287 ± 0.065	0.48 ± 0.07
areal number density of pores = (*N*/*A*)_PM_ [μm^–2^]	0.21	1.50	0.84
porosity of the porous membrane, κ_PM_^b^	0.23	0.43	0.40

a Arithmetic mean value and corresponding standard deviation.

b (Area of a pore)·(areal number density of pores).

As expected from the ellipsoidal shape of the particles, we indeed obtained elliptical pores. In the stage of composite membrane, the particles are partially embedded in a layer of cross-linked monomer of a thickness comparable to that of the particle minor axis. Due to the convex shape of the particles, the pores left behind after particle removal have concave walls; this gives rise to major and minor axes of the pores smaller than the corresponding dimensions of the templating particles. In relative terms, the dimensions of the minor axis are affected more than the dimensions of the major axis. Thus, the aspect ratios of the elliptical pores are higher than the aspect ratios of the corresponding particles.

Deviating from previous experience, the particles protrude significantly more out of the membrane toward the water phase than toward the air phase. Thus, to make the particles protrude out of the monomer air interface, we have to choose a smaller volume ratio of monomer to particle and obtain thinner membranes than usual. Unfortunately, this reduced the mechanical stability of the membrane. We tried to influence the position of the particles within the membrane by adjusting the pH value and adding surfactants but were not rewarded with success.

Further on, and deviating from previous experience^43^ as well, the porous membrane obtained after the dissolution of the particles (PM1, PM2a, and PM2b) bears pores that do not go through but still are closed by a thin “skin”. The chemical nature of this skin is not obvious to us, and Raman microscopy suggests that it might be leftovers of the polystyrene particles. However, repeated washing with good solvents for polystyrene (toluene, tetrahydrofuran) had no effect on this “skin”. Immersing the membrane in toluene for extended time periods, washing it with hot toluene, or etching it with basic or acidic solutions damaged it. Plasma etching opened the pores; however, we considered the “process window” between satisfying removal of the “skin” and damaging the remaining part of the microsieve as too narrow to be practical, especially for the microsieve with elongated pores. Closer inspections of the images of the supported porous membrane revealed that the “skin” was present in the freely suspended part of the membrane but not in the part of the membrane that was lying on the support (see for example Figure-S: 1 PM1). Most probably, capillary forces that occur during drying are stronger in the supported part than in the suspended part and rupture the “skin”. Thus, we considered removing the “skin” by applying a gentle hydrostatic pressure.

**Figure 5:**
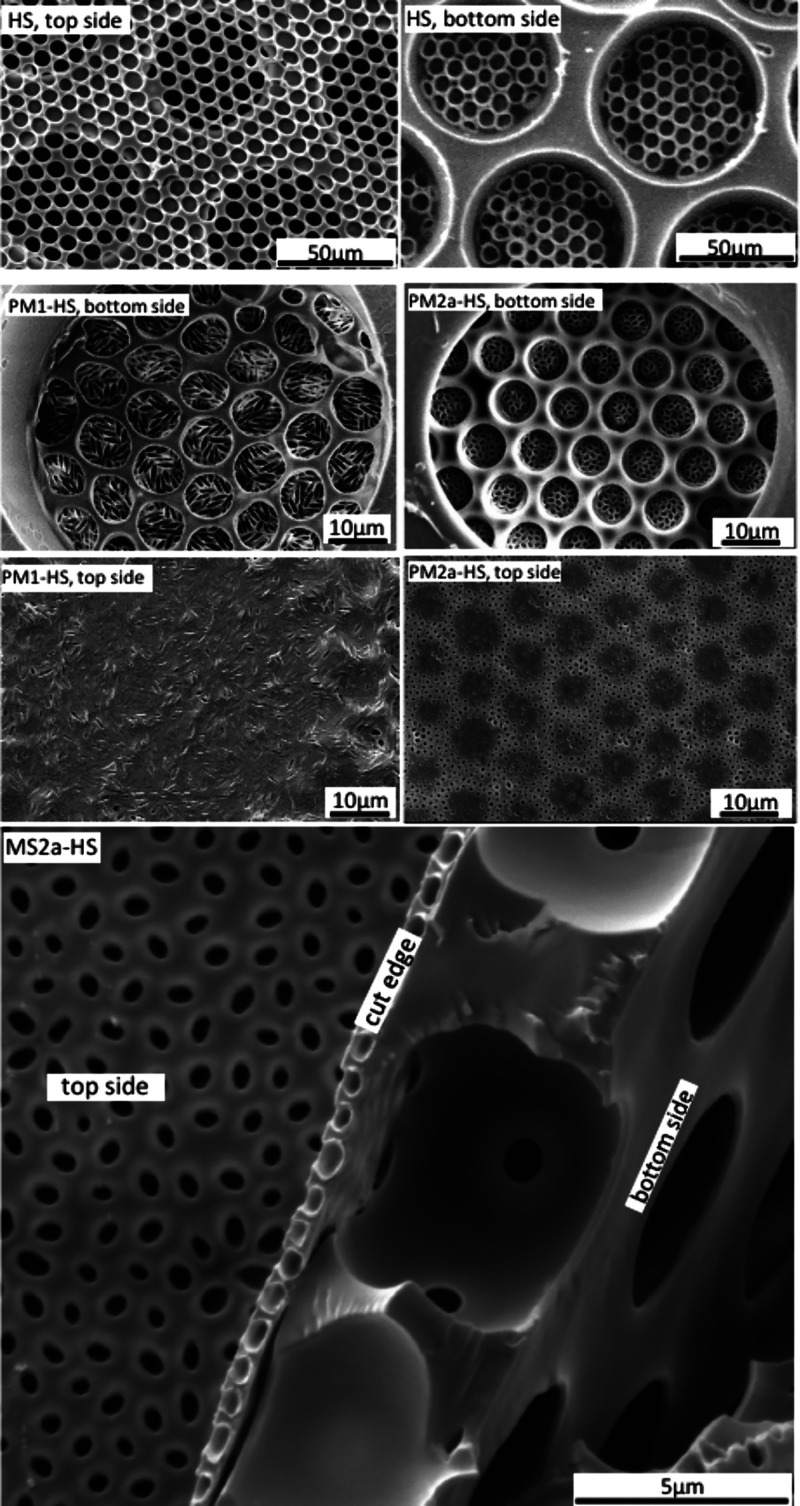
Scanning electron microscopy images of HS: hierarchical support prepared by combining a coarse and medium polymeric float-cast microsieve with circular pores of 50 and 6 μm diameter, respectively. PM1-HS, PM2a-HS: the porous membranes bearing elliptical pores (still closed by skin), supported by the hierarchical microsieve. MS2a-HS: close up of the microsieve with pores opened by applying hydrostatic pressure (for an image of MS2b-HS, see Supporting Information).

In order to do so, we prepared a polymeric hierarchical support structure (short name: HS) composed of a microsieve bearing circular through pores of approximately 70 μm diameter covered by a microsieve bearing through pores of 6 μm wide circular openings (see Figure 5, top row). We transferred the composite membranes with elliptical particles onto these hierarchical support structures (in the short name denoted by the suffix -HS), exposed them to toluene to dissolve the particles, and obtained the hierarchical structures given the short name PM1-HS and PM2a-HS, which are shown in Figure 5, second and third rows. We exposed these to a gentle hydrostatic pressure. In the case of PM1-HS, the thinner membrane with strongly elongated pores, the process window between opening the pores and damaging the membrane was too narrow. In the case of PM2a-HS, however, this treatment indeed converted the closed pores of the membrane into through pores, and we obtained the desired microsieve, given the short name MS2a-HS. An electron microscopy image of higher magnification is shown at the bottom of Figure 5, and its properties are summarized in Table 4. Unfortunately, the process window between opening the pores and rupture of the membranes was quite narrow; in approximately nine out of ten attempts, the microsieve ruptured or developed milimeter-sized defects that were visible by the unaided eye or in the microscope. With the given supply of particles, we were able to prepare a second microsieve mounted on the hierarchical support and with open pores, given the short name MS2b-HS. Its properties are listed in Table 4 and shown in the Supporting Information.

**Table 4:** Properties of the Microsieves with Elliptical Pores on Top of the Hierarchical Supporting Structure

	Short Name	
	MS2a-HS	MS2b-HS
areal number density of pores = (*N*/*A*) [m^–2^]^b^	0.4·10^12^	0.33·10^12^
porosity of the complete microsieve (on support), κ^c^	0.13	0.11
viscosity·permeance, η_water_·*Pe*_experimental_^d^ [m]	0.51·10^–9^^h^	0.57·10^–9^
	0.54·10^–9^^h^	
viscosity·permeance, theoretical [m]^e^	1.4·10^–9^	2.9·10^–9^
η_water_·⟨*V̇*_singlepore_⟩/Δ*p*, experimental [m^3^]^f^	1.28·10^–21^^h^	1.7·10^–21^
	1.35·10^–21^^h^	
η_water_·⟨*V̇*_singlepore_⟩/Δ*p*, theoretical [m^3^]^g^^,^^a^	(3.4 ± 1.7)·10^–21^	8.8·10^–21^

a Arithmetic mean value and corresponding standard deviation.

b (*N*/*A*) = κ_HS_·(*N*/*A*)_PM_.

c κ = κ_HS_·κ_PM_, κ_PM_ = porosity of the porous membrane (see Table 3), and κ_HS_ = porosity of the hierarchical supporting structure (see Table S in the Supporting Information).

d Calculated from eq 9 and η_water_ = 1.0·10^–3^ Pa s.^109^

e Calculated from eq 4 using the individual values for *a* and *b* of each pore from image analysis, *L* from Table 3, and (*N*/*A*) from Table 4.

f η_water_·*Pe*_experimental_/(*N*/*A*).

g Calculated from eq 2 using the individual values for *a* and *b* of each pore from image analysis and *L* from Table 3.

h Two succeeding experiments performed on the same area.

We measured the water permeance of 3 mm wide circular parts of the two microsieves mounted on the hierarchical support (MS2b-HS, two measurements; MS2b-HS, one measurement): At a pressure difference of approximately 10^3^ Pa (Reynolds number aprox. 0.002), the permeance of these microsieves for water was approximately *Pe* = /*A*/Δ*p* = 0.5·10^–6^ m/s/Pa, and the product of viscosity·permeance was approximately η·/*A*/Δ*p* = 0.5·10^–9^ m (Table 4 row four). These experimental values are a factor of approximately 3 to 5 lower than the theoretical values calculated from eq 4 and the properties of the microsieve obtained from image analysis (Table 4, row 5). Thus, experimental and theoretical values are in a similar range but not identical. Most probably, we did not open the pores up to the dimensions that we derived from image analysis or did not open all the pores. To compare the permeance of our microsieve to the ones that can be found in the literature, we added the corresponding data points to Figure 2 (experimental: 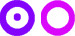; theory: 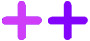). These data points are on the left side of the diagram. In other words, we successfully created a microsieve in the especially attractive regime of submicrometric pore widths. On the other hand, however, the experimental permeance is lower than the permeances of other microsieves with comparable pore width and circular pores (
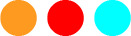
). In other words, the permeance is lower than hoped for, especially given the fact that at a given porosity and pore width theory predicts a higher permeance for elliptical pores than for circular ones. The reason for this discrepancy is not a flaw in the theory but a difference in the porosity. In the case of the inorganic microsieves symbolized by the black symbols (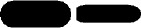 and ●), there is a difference in porosity in favor of the slit-like pores. In the case of the float-cast microsieves symbolized by the colored symbols (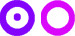 and 
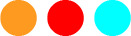
), the difference in porosity is in favor of the microsieves with circular pores. In the float-casting process, the particles arrange themselves on the water surface, laterally sightly compressed by the capillary forces exerted by the layer of liquid monomer in between them.^95^ In the case of spherical particles, this gives rise to an almost densely packed monolayer made out of a patchwork of hexagonally ordered close packed domains. In the case of the ellipsoidal particles used here, we observe an arrangement in which each particle touches mostly 5 or 6 neighbors and observe a lack of orientation correlation or positional long-range order. As a result, the lateral packing density of ellipsoidal particles and, in consequence, the porosity of the float-cast microsieve with elliptical pores is lower than in the case of circular pores. This effect increases with an increasing aspect ratio of the particles. Thus, at least with the combination of particles and monomer used here, the beneficial effects of elongated pores are, unfortunately, countered by the nonbeneficial effect of lowered porosity.

## Conclusion

We successfully used ellipsoidal particles to prepare microsieves via float-casting bearing elliptical pores with submicrometric width. We measured their permeance and compared it to literature data on other microsieves with slits and circular pores. From a practical point of view, the beneficial effects of higher permeance per pore are countered by a lower porosity.

## Data Availability

The data underlying this study are available in the published article and its Supporting Information; raw data will be made available upon request.

## Supporting Information Available

Overview images of fragments of the porous membranes (PM1, PM2a, and PM2b), images of the hierarchical microsieve used as support (HS), image of microsieve MS2b-HS, histogram of the aspect ratios of the spherical mother particles, the ellipsoidal daughter particles, and the elliptical pores in the membranes derived therefrom, and table of properties of the supporting structure (PDF)
