# Supplementary material for: Float-Cast Microsieves with Elliptical Pores
Source: Langmuir. 2024 Oct 21;40(43):22516–25. doi: 10.1021/acs.langmuir.4c01232 (PMC11526378; doi:10.1021/acs.langmuir.4c01232)
Supplement: Supplementary file 1 — la4c01232_si_001.pdf [file la4c01232_si_001.pdf]

SupportingInformation for:

Float-Cast Microsieves with Elliptical Pores

Nadine Schwaar [a], Dominik Benke [b], Markus Retsch [b,c], Werner A. Goedel [a*]

[a] Chemnitz University of Technology, physical chemistry, Straße der Nationen 62, 09116 Chemnitz, Germany, www.tu-chemnitz.de/physchem,

[*] werner.goedel@chemie.tu-chemnitz.de

[b] University Bayreuth, Department of Chemistry, Chair of Physical Chemistry I, Universitätsstraße 30, 95447 Bayreuth, Germany, www.retsch.uni-bayreuth.de; retsch@uni-bayreuth.de; [c] Bavarian Polymer Institute, Bayreuth Center for Colloids and Interfaces, and Bavarian Center for Battery Technology (BayBatt), University of Bayreuth, Universitätsstraße 30, 95447 Bayreuth, Germany

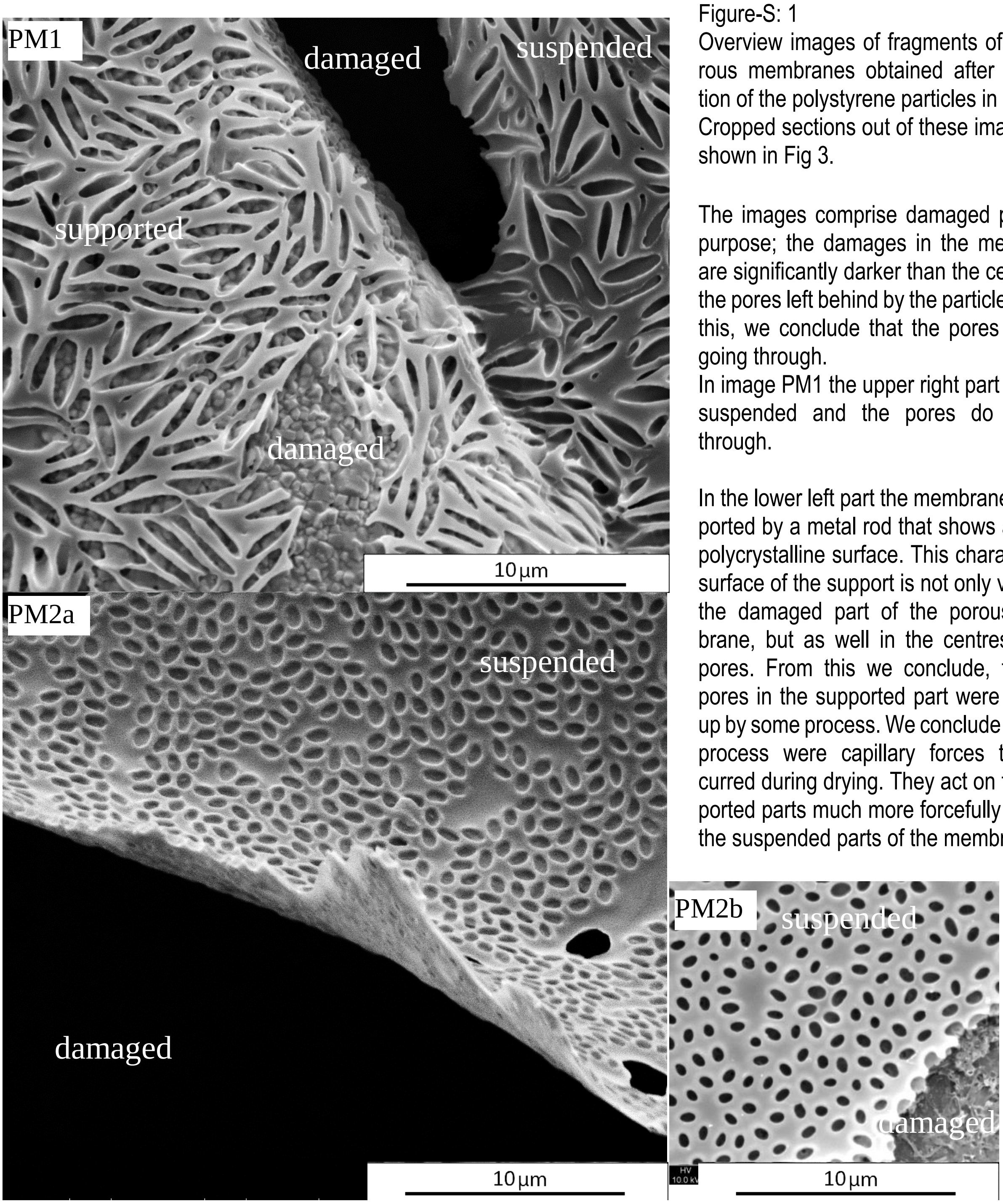

Figure-S: 1
Overview images of fragments of the porous membranes obtained after dissolution of the polystyrene particles in toluene. Cropped sections out of these images are shown in Fig 3.

The images comprise damaged parts on purpose; the damages in the membrane are significantly darker than the centres of the pores left behind by the particles. From this, we conclude that the pores are not going through.
In image PM1 the upper right part is freely suspended and the pores do not go through.

In the lower left part the membrane is supported by a metal rod that shows a grainy polycrystalline surface. This characteristic surface of the support is not only visible in the damaged part of the porous membrane, but as well in the centres of the pores. From this we conclude, that the pores in the supported part were opened up by some process. We conclude that this process were capillary forces that occurred during drying. They act on the supported parts much more forcefully than on the suspended parts of the membrane.

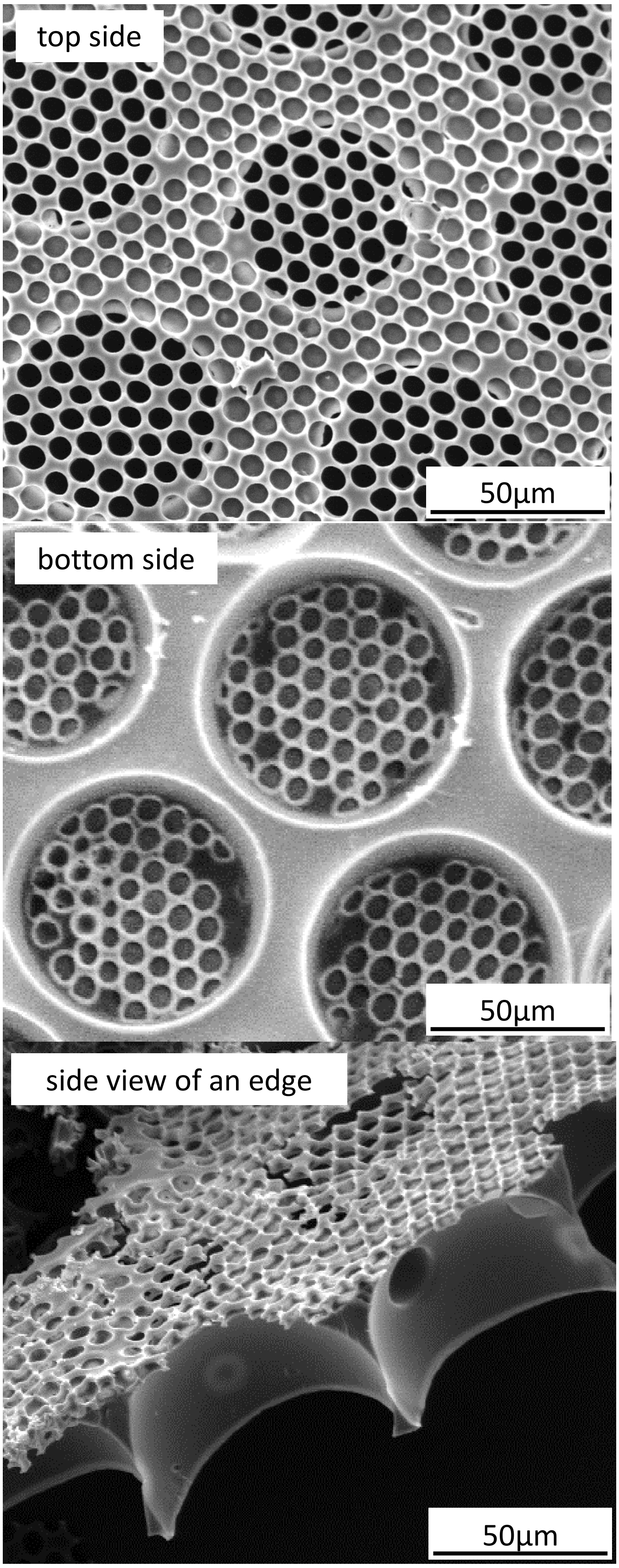

Figure-S: 2

Scanning electron microscopy images of the hierarchical microsive used as support for the porouse membrane and microsieve with elliptical pores.

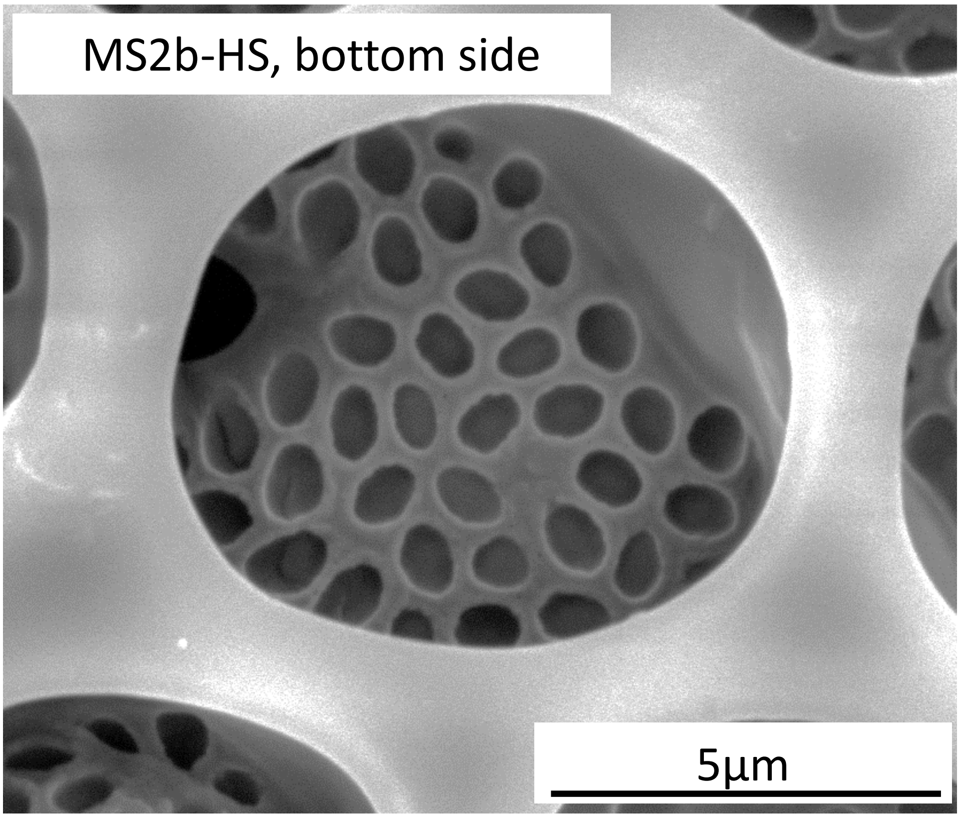

Figure-S: 3
Scanning electron microscopy images of the hierarchical microsiveMS2b-HS

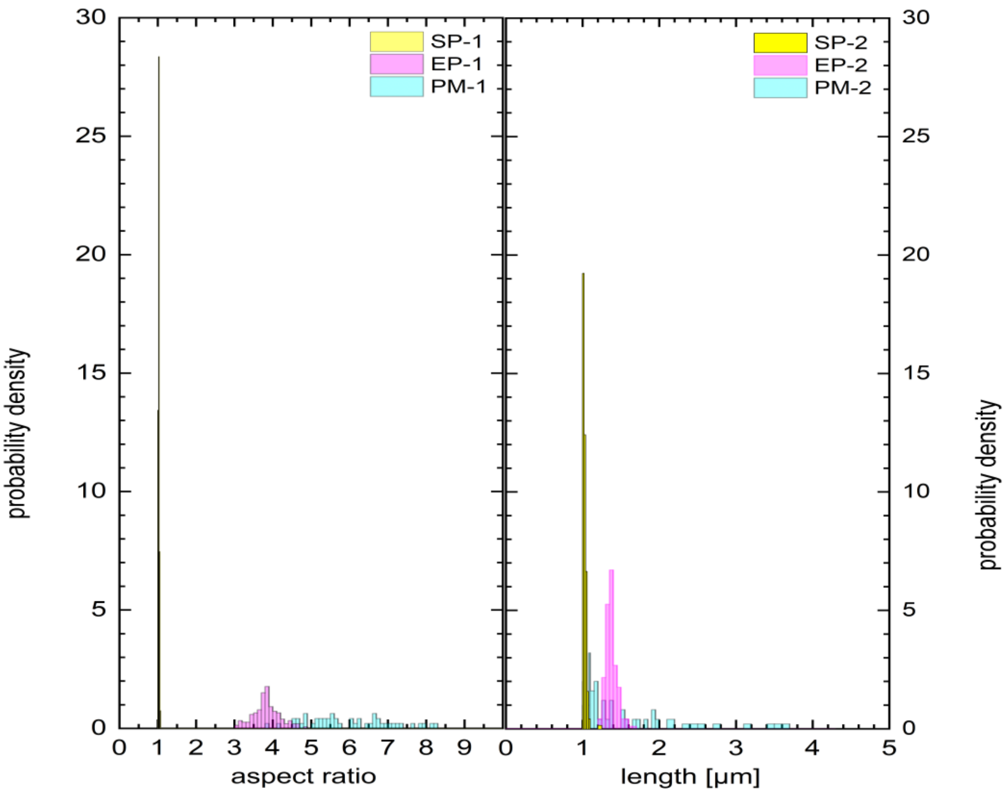

Figure-S: 4
Histogram of the aspect ratios of the spherical mother particles, the ellipsoidal daughter particles and of the elliptical pores in the membranes derived therefrom.

Table S:
Properties of the hierarchical supporting structure, Data taken from ref [40]
(for an image of the supporting structure see Fig. 5 top row)

	short name
	HS
thickness of coarse layer [µm] *)	46.0 ± 3.9
radius of a coarse pore, *rc* [µm] *)	29.0 ± 3.2.3
porosity of the coarse layer, κ_C ‡)	0.51
thickness of medium layer [µm] *)	6.01 ± 0.79
radius of medium pore, r_M [µm] *)	3.45 ± 0.33
porosity of the medium layer, κ_M ‡) *)	0.52
thickness of hierarchical supporting structure [µm]	52.0 ± 4.0
porosity of the hierarchical supporting structure, κ_{HS} #)	0.27

*) arithmetic mean value and corresponding standard deviation, ‡)= (area of a pore) · (areal number density of pores), °) $\kappa_{HS} = \kappa_C \cdot \kappa_M$
